# Declaration of common standards for the preregistration of animal research—speeding up the scientific progress

**DOI:** 10.1093/pnasnexus/pgac016

**Published:** 2022-03-16

**Authors:** Céline Heinl, Anna M D Scholman-Végh, David Mellor, Gilbert Schönfelder, Daniel Strech, Steven Chamuleau, Bettina Bert

**Affiliations:** German Federal Institute for Risk Assessment, German Centre for the Protection of Laboratory Animals (Bf3R), 10589 Berlin, Germany; Netherlands Heart Institute, 3511 EP Utrecht, The Netherlands; Center for Open Science, Charlottesville, VA, 22903-5083, USA; German Federal Institute for Risk Assessment, German Centre for the Protection of Laboratory Animals (Bf3R), 10589 Berlin, Germany; Charité-Universitätsmedizin Berlin, corporate member of Freie Universität Berlin, Humboldt-Universität zu Berlin, and Berlin Institute of Health, 10117 Berlin, Germany; QUEST Center for Responsible Research, Berlin Institute of Health (BIH) at Charité, 10178 Berlin, Germany; Department of Cardiology, Amsterdam University Medical Center, 1105 AZ Amsterdam, The Netherlands; German Federal Institute for Risk Assessment, German Centre for the Protection of Laboratory Animals (Bf3R), 10589 Berlin, Germany

**Keywords:** open science, preregistration, animal research, preclinical research, 3R, research methods

## Abstract

Preregistration of studies is a recognized tool in clinical research to improve the quality and reporting of all gained results. In preclinical research, preregistration could boost the translation of published results into clinical breakthroughs. When studies rely on animal testing or form the basis of clinical trials, maximizing the validity and reliability of research outcomes becomes in addition an ethical obligation. Nevertheless, the implementation of preregistration in animal research is still slow. However, research institutions, funders, and publishers start valuing preregistration, and thereby level the way for its broader acceptance in the future. A total of 3 public registries, the *OSF registry*, *preclinicaltrials.eu*, and *animalstudyregistry.org* already encourage the preregistration of research involving animals. Here, they jointly declare common standards to make preregistration a valuable tool for better science. Registries should meet the following criteria: public accessibility, transparency in their financial sources, tracking of changes, and warranty and sustainability of data. Furthermore, registration templates should cover a minimum set of mandatory information and studies have to be uniquely identifiable. Finally, preregistered studies should be linked to any published outcome. To ensure that preregistration becomes a powerful instrument, publishers, funders, and institutions should refer to registries that fulfill these minimum standards.

## Preregistration Speeds Up the Scientific Progress in Biomedical Research

Robust and reliable research is key to make the scientific output trustworthy for science, industry, and for the public. Preregistration is a straightforward option to improve the reliability and validity of published research (1). To preregister a study, scientists have to determine a detailed research plan before starting their experiments. Thereby, the number of studies conducted over a period of time will become visible, regardless of their outcome or whether the results are published. If a preregistration also includes a detailed analysis plan, it will further clarify the distinction between a priori hypotheses and unplanned analyses. This way, questionable research practices, such as p-hacking and HARKing (hypothesizing after results are known) that too often jeopardize the validity of published data, will effectively be prevented (2, 3). In addition, preregistration can increase awareness for specific steps that can be implemented to reduce the risk of bias, such as blinding and randomization. By creating transparency in the research process, preregistration can further increase trust in published studies.

Preregistration is already recommended by the Declaration of Helsinki for all clinical studies and is mandatory by law for most regulated clinical trials (4). It took many years and required the involvement of funders, publishers, and policymakers to make preregistration a scientific routine for clinical trials. Moreover, preregistration gained rising attention in disciplines like psychology and economy (5). However, it remains nearly absent in fundamental biomedical research.

It has to be kept in mind that a vast part of preclinical research relies on animal experiments. Since the translation of preclinical successes often fail in clinical trials, especially the quality of animal studies was repeatedly questioned (6). Thus, animal testing is an ethical challenge that is still controversially discussed. Animal experiments are generally accepted under the basic assumption that they will produce new knowledge for our society and might contribute to medical progress or ensure human safety. Despite these provisions, recent studies indicate that a significant number of animal experiments is never published (7, 8). Performing animal experiments without sharing gained information strongly contradicts ethical principles as these animals do not contribute to any scientific advancement.

It could be argued that current publication conventions come at a clear cost to animal welfare. Indeed, researchers are rewarded for statistically significant, surprising, and novel findings by the publication system in place. Thus, results not fitting the story often end up in the drawer. This selective reporting of animal experiments can induce the unaware doubling of experiments in different labs. It has to be acknowledged that null or negative findings also contribute to the gain of scientific knowledge. Furthermore, insufficient quality of studies or incomplete reporting can eliminate or bias the respective knowledge gain (9). Animal lives are wasted when the respective experiments are lacking any benefit for our society (10). It, thus appears to be an ethical duty to maximize the impact of performed animal studies by sharing all gained information, including data and optimizing the validity of the outcome by thorough planning. Sharing data has been shown effective in increasing the output as the same data can be analyzed by different research groups and scientists can work on larger sample sizes by combining multiple data sets (11). Shared datasets open-up new possibilities for statistical analysis and can help optimizing the data collection. Indeed, taking into account measurement reliabilities that can be assessed from published data when designing a new study can further improve the translation of published outcomes (12, 13). Improving rigor by choosing the most appropriate statistical models such as considering using mixed-effect models when adequate over the commonly used ANOVA could further enhance the validity of the results (14, 15). Preregistration templates that are consulted in the planning stage of a project could be an ideal way to support scientists in developing an optimal statistical framework for their study by raising researcher's attention on best practices.

We are convinced that preregistration of animal studies could not only speed up medical progress by improving quality and reporting of biomedical research, but could also prevent further waste of animal lives by bringing unfitting findings to light.

## The Lack of Preregistration in Biomedical Animal Research

Despite the advantages for the global improvement of scientific progress, the uptake of preregistration in animal research is still slow (16, 17). Due to the very competitive nature of biomedical research, scientists might hesitate to endorse open science practices, fearing a disadvantage over their research fellows. For example, prospective study registration is associated with lower reported effect sizes (18–20). This relationship suggests that preregistration accomplishes 1 of its tasks, of reducing flexible data analyses that inflate effect sizes. Individuals voluntarily preregistering their study may, thus be more modest in their effect sizes than their peers. The present scientific system does not sufficiently value and reward this effort that will help show validity and trustworthiness of the scientific work and is worth the bureaucratic burden (21).

Besides the reservations toward preregistration existent in all disciplines, some hesitations might be specific to animal research. Indeed, the attitude of secrecy that long time persisted in all aspects of animal experimentation could further explain the doubts toward preregistration. Fearing consequences from public opinion or authorities involved in the legal process of animal experimentation, researchers prefer avoiding risks arising from open science (22). However, a change of attitude can be observed as more and more scientists call for the transparent communication of animal experiments (23).

Beyond these deeply rooted problems, 1 possible reason for the low participation is that an important part of animal scientists simply is still not aware of the possibility of preregistering their preclinical research, and thus does not know the benefits of preregistration for their work. Preregistration can assist researchers in planning their study thoroughly by asking detailed questions concerning the study protocol. This information is often needed when publishing the study results. Preregistration can also raise the awareness of common mistakes, such as not reporting the method of randomization, blinding, or sample size calculation. Contrary to many fears, preregistration actually protects the intellectual property, by providing a timestamp for the research idea.

## A Change of Attitude Toward Preregistration in Animal Research

Scientific literature is flooded by positive findings (24). However, science has to acknowledge that null and negative results also contribute to the knowledge gain. Scientists should be more open about things going wrong and publishers should provide the opportunity to publish failures. Key for both measures is that quality and transparency in research needs to be rewarded. By preregistering a study, scientists show their commitments to these values. This should be considered from institutions deciding on new positions, from funders when awarding grants, and from journals when reviewing manuscripts (25, 26). Targeted educational actions, reaching scientists at all stages of their career, should be established to spread information about preregistration.

Within the last years, a change of attitude toward preregistration can be observed (27). Indeed, publishers start to include preregistration of animal studies in their publication guidelines (28), funders ask for preregistration as a prerequisite for grant application (29), and policymakers discuss preregistration in animal research (30). An Advisory Committee to the Director (ACD) working group advising the National Institute of Health (NIH) on enhancing rigor, transparency, and translatability in animal research has recommended to launch a pilot study to further promote and evaluate the benefits of preregistration of animal research (31). A growing number of universities include open science education in their curricula or evaluate candidates for new positions on their contribution to open science and not on h-index or impact factor (32, 33). These measures could have a powerful impact on preregistration in biomedical research in the long run.

## Finding the Right Preregistration Type For Your Research

Scientists already have different possibilities to preregister their animal studies. They can opt for a preregistration bound to a certain journal—so called registered report—or choose the preregistration in a public registry.

Hundreds of journals already publish registered reports, including many journals open to animal research (34). Registered reports pass 2 steps of peer-review. The first peer-review will decide on the idea and study plan that are submitted to a journal before scientists start their experiments. After an “in-principle-acceptance” researchers can conduct their study as planned. If the authors conduct the experiments as described in the accepted study protocol, the journal will publish the outcome of the study independent of its results.

In addition, several public online platforms were launched over the last years, enabling the preregistration of animal research in a more autonomous way. These public registries are independent of a journal and allow full flexibility in the preregistration process. Scientists can freely decide whether they register complete studies, pilot studies, or only some experimental groups that will later be combined in a manuscript. This makes public registries suitable for hypothesis testing as well as hypothesis generating research.

The open science framework (OSF) was the first platform that allowed preregistration of studies from different disciplines. The *OSF registry* gives the option to choose between different preregistration templates or to preregister a study without using any template. The *OSF registry* is embedded in a framework of various open science project management tools. Thereby, all the steps of a research project can transparently be mapped. This includes the option of sharing data, analysis code, preprints, and even talks or posters. In contrast, *preclinicaltrials.eu* and *animalstudyregistry.org* are pure registries. Prerequisite for the preregistration for both platforms is the use of animals in the planned study. *Preclinicaltrials.eu* and *animalstudyregistry.org* encourage the preregistration of basic exploratory research as well as confirmatory preclinical studies.

The organizations hosting the registries are diverse. The *OSF registry* is a platform built and maintained by the Center for Open Science, a nonprofit technology organization founded in 2013 receiving funding from individuals, philanthropic foundations, and US federal agencies (35). *Preclinicaltrials.eu* is embedded in academia, and is partly supported financially by the Dutch government. It was launched in 2018 and is hosted by the Utrecht University and endorsed by animal welfare bodies, open science platform, and several grant suppliers (36, 37). *Animalstudyregistry.org* followed in January 2019 (38). It is an initiative of a governmental institution in Germany, the German Centre for the Protection of Laboratory Animals (Bf3R), which is part of the Federal Institute for Risk Assessment (BfR). The 3 registries differ in their provided options for the preregistration of studies. The embargo-period, during which details on the study will remain hidden for the public, varies across the registries. Whereas *preclinicaltrials.eu* offers an embargo-period for up to 1 year (which can be extended upon request), *OSF registry* supports a duration for up to 4 years and *animalstudyregstry.org* for up to 5 years until the study becomes completely publicly accessible. *Preclinicaltrials.eu* and *animalstudyregistry.org* ask for entries in English whereas the OSF is not restricted to any language. After final registration, studies cannot be withdrawn from *preclinicaltrials.eu*. Researchers can retract studies in the *OSF registry* and, with good reason accepted by the support team, also in the *animalstudyregistry.org*. Retracted studies will then only leave metadata in the respective registry. The *OSF registry* and *animalstudyregistry.org* offer the possibility to upload files with the preregistration of a study.

The information required to preregister a study varies between the registries. Preregistration templates with specific questions can support scientists in planning their study and allow preregistration to develop its full strength (39). All templates cover information on the study design, the study status, and the statistics. *Animalstudyregistry.org* and *preclinicaltrials.eu* further have fields for the planned methods. This might help other scientists later to track methodological changes occurring during the research process, which are often missing in the final publications and might have high practical relevance for colleagues in the field. In addition, *preclinicaltrials.eu* and *animalstudyregistry.org* include questions in their form on the characteristics of the animals used.

To ensure the quality, transparency, and security of preregistrations, common minimum standards for preregistration in biomedical research need to be defined. These quality standards are met by the 3 registries and should be considered for newly emerging preregistration platforms.

## Common Goals—Common Standards

All registries are aiming for a significant improvement of research quality and transparency. Likewise to clinical registries, common standards for these and future registries should be defined to ensure that preregistration becomes and remains a valuable tool in preclinical research (40). These standards include infrastructural requirements and mandatory contents for preregistration templates. We identified 8 requirements that a registry should fulfill to effectively improve research quality:

### Publicly accessible for free

To ensure transparency, all entries to a registry should be publicly accessible with no charges for authors or users of the platform, with the option of an embargo period.

### Transparency on ownership and financial sources

Preregistering a study in a public registry is an act of trust that needs to be valued by ensuring high standards of transparency on the organization hosting the platforms. This includes the full disclosure of funding sources.

### Possibility to track changes

Each preregistration should receive a time stamp. All changes occurring after registration of the study need to be distinguishable from the original preregistration and ideally receive further time stamps.

### Data security

In order to affirm trust in preregistration, all organizations hosting a registry have to provide a safe data storage. This point is most important when preregistered studies are still under embargo, meaning that the main content is not publicly accessible.

### Sustainability of data storage

Since preregistration is a long-term process, the sustainability of the data storage needs to be ensured. Registration platforms need to provide an adequate IT infrastructure to prevent corruption or loss of data and the assurance that preregistered studies will remain accessible within the next decades.

### Citability of the preregistration

Preregistration has to become a benefit for scientists. To demonstrate the efforts authors performed to improve the quality and transparency of their research, studies have to be uniquely identifiable (e.g. DOI or other persistent, unique identifier) to make them citable in submitted manuscripts or grant applications. Citability also implies persistence, meaning that authors cannot remove content without a withdrawal process that leaves a record.

### Link between preregistration and outcome

Preregistration unfolds its full effect when the planned study can be compared with its outcome. It will, thus be crucial to provide the possibility to link the preregistered plan with any resulting data or publications.

### Minimal content of a preregistered study

Even though the fields of a preregistration template can differ, essential information in the preregistration has to be covered. Preregistration should include following mandatory information to make it a useful tool. A title and the name of the author have to be provided. Scientists should clarify whether the study already started at the time of preregistration. Clearly stating the hypothesis and defining the type of research are critical. The following items on the study design may not be required for all preregistrations, but serve important roles in minimizing questionable research practices. Basic information on good scientific practices, like blinding or randomization, is essential. Defining analysis details such as the variables, exclusion and inclusion criteria, stating the sample size together with a short rationale for it as well as the planned statistical analysis can further effectively prevent p-hacking and HARKing. For animal experiments, it should be mandatory to declare the species, the strain, and the sex of the animals. Likewise, describing the animal models or the methods used on the animals is key (see also Table 1).

**Table 1.** Optimal content required for an effective preregistration.

OPTIMAL content for a preclinical preregistration	
1. General information	These should at least include a title and the name and affiliations of the authors.
2. Study status	It has to be clear whether experiments have been started or if data already existed before preregistering the study.
3. Hypothesis	Clearly stating the hypothesis will help to differentiate between exploratory and confirmatory research.
4. Study design	Describing the study design, including basic information on blinding and randomization, will be critical.
5. Methods	A detailed description of the methods will help to transparently trace the research process and prevent the dropout of practical knowledge that could be useful for other scientist working in the same field.
6. Statistics	Defining the variables and stating the sample size with a concise explanation, the experimental unit, and all parts of the statistics that were planned a priori is central to later judge the validity of the outcomes.
7. Animals	Authors should declare the species, the strain, and the sex of the animals they will use and describe all methods used on the animals.

## The Future of Preregistration in Animal Research

Research quality and transparency have to become a new value that needs to be rewarded by institutions, funders, and journals. With respect to animal experiments, preregistration also addresses the ethical aspect of transparency in biomedical research. Preregistration, together with the complete reporting of results, can reduce the repetition of unnecessary experiments, and thereby avoid the waste of animal lives.

Preregistration in preclinical research involving animals gains rising attention. We can expect that new preregistration platforms will evolve. We recommend they should fulfill our defined minimum standards to advance the quality and transparency of biomedical research. Beyond the 8 criteria stated above, some optional points could help a broader acceptance of preregistration in animal research. This could include offering an embargo-period that responds to the widespread fear within the scientific community of the theft of ideas. In addition, covering all points of the ARRIVE (Animal Research: Reporting of In Vivo Experiments) guidelines in the preregistration form could help to reduce the administrative burden for scientists. The ARRIVE guidelines define essential details of in vivo animal research that need to be reported in a publication to enable a correct interpretation of the data. Since their first publication in 2010, they were already endorsed by over 1,000 journals and updated in 2020 (41, 42). Scientists are repeatedly asked for similar details on their experiments during the application for animal study protocols, for the preregistration of a study, or for a checklist from journals when submitting a manuscript. Harmonization between these questionnaires could facilitate the reuse of collected information. Furthermore, a verification of the preregistered records performed by competent personnel from the registry could further ensure the plausibility and completeness of the minimal content demanded from the preregistration template. However, preregistration registries do not aim to act as an ethics committee and will not judge the validity of the content. These verifications should target solely completeness (in the level of detail), authenticity (e.g. ensure that it is a prospective registration), and potential error or misplacement (e.g. misunderstanding regarding what is requested and information being misplaced). Reviewers or the scientific community will then evaluate the quality of the contents. These 3 points might not directly affect the quality of the preregistrations, but might contribute to a wider acceptance of preregistration by scientists that is essential for its global beneficial effect on biomedical research.

Preregistration can only prevail when entries follow the FAIR principle, i.e. they should be Findable, Accessible, Interoperable, and Reusable. It is, thus conceivable that 1 day a searchable database with entries from all different registries will improve the visibility and the scope of preregistration. To fulfill this prerequisite, it will be essential that registries will offer an application programming interface (API) that other online tools could use. An API could help to connect other open science tools, like electronic lab books or preprints with the registries, which would significantly simplify the practicability of preregistration and help scientists to integrate it in their scientific routine.

Collaborations between the platforms, like for the present declaration of minimum standards, could further advance preregistration. Platforms may in future regularly exchange experiences to better adapt the databases according to the needs of the users or adjust the communication efforts aiming to spread preregistration among scientists. Joining forces further puts platforms in a stronger position when it comes to negotiations with stake holders. Indeed, to ensure that these defined minimum standards prevail in the future, it will be decisive that publishers, funders, and institution that state preregistration as a requirement or recommendation refer to preregistration platforms that guarantee these standards.

## Notes

**Competing Interest:** Céline Heinl, Gilbert Schönfelder, and Bettina Bert: employed at the German Federal Institute for Risk Assessment and part of the German Centre for the Protection of Laboratory Animals (Bf3R), which developed and hosts *animalstudyregistry.org. Anna M.D. Scholman-Végh: works as a daily director of preclinicaltrials.eu*.Steven Chamuleau: member of the steering committee of*preclinicaltrials.eu*.David Mellor: employed by the nonprofit Center for Open Science, which runs the Open *Science Framework*. Daniel Strech: no competing interests declared.
